# Challenges for nursing education in Angola: the perception of nurse leaders affiliated with professional education institutions

**DOI:** 10.1186/1478-4491-11-33

**Published:** 2013-07-17

**Authors:** Leila Maria Marchi-Alves, Carla A Arena Ventura, Maria Auxiliadora Trevizan, Alessandra Mazzo, Simone de Godoy, Isabel Amélia Costa Mendes

**Affiliations:** 1 University of São Paulo at RibeirãoPreto College of Nursing, Av. dos Bandeirantes, 3900, RibeirãoPreto, SP, Brazil

**Keywords:** Nursing workforce, Nursing education, Human resources, Nursing human resources, Palavras-chave: força de trabalho em enfermagem, Educação em enfermagem, Recursos humanos, Recursos humanos de enfermagem

## Abstract

**Background:**

Angola is one of the African countries with the highest morbidity and mortality rates and a devastating lack of human resources for health, including nursing. The World Health Organization stimulates and takes technical cooperation initiatives for human resource education and training in health and education, with a view to the development of countries in the region. The aim in this study was to identify how nurses affiliated with nursing education institutions perceive the challenges nursing education is facing in Angola.

**Methods:**

After consulting the National Directory of Human Resources in Angola, the nurse leaders affiliated with professional nursing education institutions in Angola were invited to participate in the study by email. Data were collected in February 2009 through the focus group technique. The group of participants was focused on the central question: *what are the challenges faced for nursing education in your country?* To register and understand the information, besides the use of a recorder, the reporters elaborated an interpretative report. Data were coded using content analysis.

**Results:**

Fourteen nurses participated in the meeting, most of whom were affiliated with technical nursing education institutions. It was verified that the nurse leaders at technical and higher nursing education institutions in Angola face many challenges, mainly related to the lack of infrastructure, absence of trained human resources, bureaucratic problems to regularize the schools and lack of material resources. On the opposite, the solutions they present are predominantly centered on the valuation of nursing professionals, which implies cultural and attitude changes.

**Conclusions:**

Public health education policies need to be established in Angola, including action guidelines that permit effective nursing activities. Professional education institutions need further regularizations and nurses need to be acknowledged as key elements for the qualitative enhancement of health services in the country.

## Abstract (pt)

**Abstract:**

**Introdução**

Angola é um dos países africanos que apresenta os maiores índices de morbimortalidade e devastadora insuficiência de recursos humanos na área de saúde, incluindo a enfermagem. A Organização Mundial da Saúde estimula e implementa iniciativas de cooperação técnica para a formação e aperfeiçoamento de recursos humanos na área da saúde e educação, visando o desenvolvimento dos países africanos. Este estudo teve como objetivo identificar as percepções dos enfermeiros vinculados a Escolas de Formação em Enfermagem em relação aos desafios para o ensino de enfermagem em Angola.

Após consulta ao Diretório Nacional de Recursos Humanos de Angola os líderes em enfermagem vinculados a Escolas de Formação em Enfermagem do país foram convidados a participar do estudo por meio de convites eletrônicos. Para a coleta de dados, realizada no mês de fevereiro de 2009, foi utilizada a técnica de Grupo Focal. O grupo de participantes foi direcionado para a questão central: *quais os desafios encontrados para a educação em enfermagem no seu país?* Para o registro e compreensão das informações, além do uso de um gravador, um relatório interpretativo foi elaborado pelos relatores. Os dados foram codificados via análise de conteúdo.

**Resultados**

Participaram do encontro 14 enfermeiros, sendo a maioria vinculada a escolas de ensino de enfermagem de nível técnico. Verificou-se que os líderes de Escolas de Nível Técnico e Superior de Enfermagem em Angola enfrentam muitos desafios, relacionados principalmente à falta de infra-estrutura, ausência de recursos humanos capacitados, problemas com a burocracia para a regularização das escolas e escassez de recursos materiais. Em contrapartida, apresentam soluções centradas predominantemente na valorização dos profissionais de enfermagem, o que implica em mudanças culturais e de atitude.

**Conclusões**

É necessário o estabelecimento de políticas públicas de educação em saúde em Angola com diretrizes de ação que permitam a atuação efetiva dos enfermeiros. Salienta-se a necessidade de regularização das Escolas profissionalizantes e o reconhecimento do enfermeiro como elemento chave para o incremento da qualidade dos serviços de saúde do país.

## Background

The mission of the World Health Organization [1] is health promotion, focusing its guidelines on strategic regions, including the African continent, which is characterized by high morbidity and mortality rates and a low human development index (HDI). Angola, a Portuguese-speaking African country, was devastated by a long-lasting civil war, resulting in a low HDI, which has ranked the country in the 148th position on the list of countries included in the Human Development Report of the United Nations Development Program [2].

One of the causes of the high morbidity and mortality rates is the lack of human resources. According to the Global Health Workforce Alliance [3], in 57 countries around the world, less than 23 health workers are available for every 10000 people. As a result, maternal and child mortality ratios in all of these countries surpass those measured in developed countries, even reaching extremely alarming levels. Angola is part of this group, with one physician and 63 nursing professionals for every 10000 inhabitants and 220 deaths of children before the age of five for every 1000 live births.

With a view to promoting development in African countries, WHO and other international non-governmental organizations stimulate and take technical cooperation initiatives in health and education, valuing human resource education and training [1,4]. The Task Force on Health Systems Research mentioned human resources for health as a priority to achieve the Millennium Development Goals (MDG) [5]. In addition, according to the Millennium Development Goals Report [6], the global achievement of the MDGs will only be possible through cooperation, strengthening of partnerships between national and international authorities and political commitment to nations. It is fundamental for those in power to prioritize the interventions that are most influential or capable of exerting a cascade effect on a wide range of indicators. The intrinsic connection among gender empowerment, education, poverty and health is well known. These and other relations need to be further explored with a view to sharpening the intervention’s strategic focus, aiming for the development of countries devastated by misery.

In 2010, Brazil and Angola formalized their interest in reinforcing their exchange relations in the fields of technical-scientific knowledge, defenceand human resource education. In the Joint Declaration for a Strategic Partnership, several bilateral agreements were closed, including Technical, Scientific and Technological Cooperation, which comprises the strengthening of qualified human resource development and joint bilateral research promotion, in areas like health, education and higher and postgraduate teacher training [7]. The common language is a facilitator to broaden the relations between both countries and favours the expansion of cooperation agreements in professional education and training for nursing teaching and care delivery.

Among the 45 WHO Collaborating Centres for Nursing and Midwifery Development, only one is hosted in a Portuguese-speaking country,Brazil, represented in this global support network by the University of São Paulo at RibeirãoPreto College of Nursing. Since 1988, the college has served as a WHO Collaborating Centre for Nursing Research Development. Therefore, the institution has occupied the forefront in movements and programmes to respond to nursing human resource needs in Portuguese-speaking African countries(PALOP).

The aim in this study was to identify how nurses affiliated with nursing education institutions perceive the challenges nursing education is facing in Angola. Knowing the opinion of nurse leaders in Angola about relevant elements for nursing education is important because they are directly involved in decision-making processes involving nursing teaching and care practice in that country. They are responsible for discussing changes with all stakeholders involved, as well as for assessing the circumstances, values and risks of each alternative and for identifying the needs and consequences of each action, with a view to facilitating teaching and promoting the growth and recognition of Angolan nursing.

## Methods

In this qualitative study, the focus group technique was applied to collect the data. This technique is characterized by the discussion of different aspects related to the same theme, permitting a more in-depth analysis of problems, based on the participants’ perceptions, feelings and emotions [8,9].

After consulting the National Directory of Human Resources in Angola, the nurse leaders,who are professionals affiliated with professional nursing education institutions in the country, were identified. Then, directors of technical and higher education institutions were invited by email to participate in a meeting. The invitations contained superficial information about the research theme, to avoid them from bringing any prejudiced ideas on the theme to the meeting. The main limitation to their participation was their non-availability to take part in the focus group on the established date and time.

The focus group dynamics took place in February 2009, between 8 a.m. and 10 a.m., in the auditorium of a higher education institution located in the province of Luanda. The meeting consisted of the following phases: organization of the room to hold the dynamics, welcoming of the participants, explanations about the technique, reading and signing of the informed consent form, presentation of the participants, start of the guiding questions, and synthesis of the main themes addressed.

The researchers were divided into the roles of coordinator, moderator and reporter and the participants were informed about each person’s role. A time was preset for the activities to end.After the participants had presented themselves, the group was oriented towards the central question: what are the challenges faced with regard to nursing education in your country?

To register and understand the information, the reporters used a recorder and elaborated an interpretative report. The testimonies were subject to exhaustive reading, followed by the organization and categorization of the data by highlighting recurring themes or patterns. The analysis categories led to the following descriptive themes: challenges for nursing education and solutions to overcome the challenges.

At the end, the data were interpreted, emphasizing the numerical description of how certain explanatory categories figured in the discussions [10]. Under the flexible guidance of the moderator, who guaranteed that the participants continued focusing on the theme, a number of challenges and solutions were proposed.

This study received approval from the Research Ethics Committee at the University of São Paulo at RibeirãoPreto College of Nursing. Respect for the participants’ dignity and autonomy were guaranteed. Their identities were preserved and their privacy was respected in all research phases, recognizing their vulnerability and free will. They were guaranteed the freedom to cease their participation at any time, without any kind of penalty if they refused to participate or decided to drop out of the study. The research collaborators complied with the ethical determinations of Resolution 196/96 by the Brazilian National Health Council, regarding the authorization to record the participants’ discourse and the signing of an informed consent form, and also signed a group contract to guarantee shared ethical secrecy and facilitate the interaction process. The participants were informed that recording their statements was necessary to guarantee the reliable transmission of the contents, but that these recordings would solely be used for research purposes and destroyed at the end of the study. In order to preserve the participants’ identity and anonymity, capital letters were used in the transcriptions to identify them.

The institutions of origin were asked to provide logistic support and grant permission for the nurses’ participation; the researchers funded two days of accommodation for each participant at the data collection site.

## Results

Fourteen nurses participated in the meeting, eight of whom were affiliated with technical schools, four with higher education institutions and two with the Ministry of Health of the Republic of Angola. The participants came from five provinces, including the capital Luanda. Two nurses indicated they were unable to participate in the study, due to professional commitments that prevented them from travelling to the place of data collection on the date set for the activity. The majority (64.3%) of nurses were between 51 and 55 years of age, with ten (71.4%) male and four (28.6%) female participants.In Table 1, the participants’ profile of professional education, function and experience in the job is displayed.Among the participants, the nine nurses who were active in teaching accumulated other roles also listed in Table 1.

**Table 1:** Participants’ profile, considering professional education, role and experience in the current role

	**Number**	**%**
**Professional education**		
Undergraduate degree in nursing	8	57.1
Specialization degree in nursing	1	7.1
Master’s degree in nursing	4	28.6
Doctoral degree in nursing	1	7.1
**Role**		
Teaching	9	64.3
Unit management	5	35.7
Coordination of scientific/pedagogical activities	4	28.6
Coordination of courses/services	2	14.3
Department head	2	14.3
Technical advice	1	7.1
**Experience in the current role**		
1 to 5 years	7	50
6 to 10 years	4	28.6
11 to 15 years	2	14.3
16 to 20 years	1	7.1

Angola, 2009.

First, the group discussed the different kinds of challenges experienced in nursing education in Angola, as shown in Table 2.

**Table 2:** Challenges experienced in nursing education in Angola

**Challenges**		**Number (%)**
1	Disequilibrium between supply and demand for professional education places	13 (92.9)
2	Financial aspects	
	Lack of funding	12 (85.7)
	Lack of autonomy for resource management	10 (71.4)
3	Physical structure	
	Inappropriate and/or insufficient physical structure	10 (71.4)
	Lack of maintenance and preservation of physical areas	9 (64.3)
	Central location of schools in the capital	9 (64.3)
	Lack of logistic support to receive guest lecturers	8 (57.1)
	Lack of public training areas	8 (57.1)
4	Human resources	
	Education abroad does not attend to Angola’s needs	8 (57.1)
	Lack of teachers	7 (50)
	Lack of teacher training	7 (50)
	Inappropriate work conditions	7 (50)
	Subordination of nursing	6 (42.9)
5	Gaps in nursing curriculum	
	Predominant focus on hospital area	7 (50)
	Early professional activities	6 (42.9)
	Education does not distinguish among professional categories	5 (35.7)
6	Legal aspects	
	Lack of regulations for Technical and Vocational Schools in Health	10 (71.4)

The participants mentioned the insufficient number of places to respond to the demand for secondary and higher nursing courses. One of the causes for this lack of places is related to the displacement of nursing schools to make room for medical schools. In addition, most educational and training institutions are located in the capital, while few schools are located in the provinces, making it difficult for most potential students to have access.

Among the financial issues that further enhance these difficulties, payment requirements to use training areas in private institutions were mentioned, equivalent to 20 dollars per student/day. Furthermore, professionals from these services demand payment to monitor the students in the field.

For a number of reasons, the Republic of Angola counts on expertise established in other countries to prepare and train qualified professionals, but one of the factors attributed to the lack of human resources relates to the trend for Angolan students to remain in the countries where they received their education, with low rates of return after graduation. In addition, the skills gained abroad do not always correspond to the local reality and needs.

The insufficient number of nurses with teacher training leads to the use of non-qualified staff to supervise practical activities in clinical training areas. The participants reported that most professionals active in this sphere only hold a primary education degree. Special training programmes in nursing are unable to function on a regular basis, due to a lack of trained teaching staff, which exacerbates the lack of places even further.

Among the challenges mentioned, work conditions in public institutions were highlighted, which as opposed to the private sector, do not favor good professional performance. Thus, some professionals migrate to private services, in search of more appropriate work conditions to perform their functions.

The matter that the group felt most strongly about was the need to regulate the Technical and Vocational Schools in Health (ETPS). The participants reported that the Secondary Education Institutions in Health (IMES) have always offered inappropriate training to technical professionals, but that proposals have been formulated to transform the IMES into ETPS; no political support has been found for this initiative though.

Another aspect addressed was the teaching focus, which privileges tertiary care, in the IMES as well as in Higher Education Institutions (HEI), which does not correspond to or only partially attends to market and community needs. The insufficient number of hospital institutions with human and material resources at their disposal further aggravates this difficulty, mainly at Intensive Care Units and Operating Rooms.

A curriculum revision is due at the secondary and higher education levels, in view of the inappropriate similarity between both programmes. In the same sense, another source of concern is the focusof the undergraduate course curriculumon preparing students for professional activities in the third year of their programme, while only graduating as a registered nurse at the end of the fourth year.The participants consider that the lack of sensitivity about nursing human resource training in Angola among those in power, across all activity spheres, further aggravates this situation.In Table 3, the solutions the group proposed are displayed, related to the challenges mentioned.

**Table 3:** Solutions proposed by research participants in view of the challenges faced in nursing education in Angola

**Solutions**	
1.	Resource decentralization/financial autonomy
2.	Elaboration of cooperation protocols between health schools and services
3.	Creation of a university hospital
4.	Regularization of Technical and Vocational Schools in Health (ETPS)
5.	Establishment of valuation policy for nursing professionals
6.	Attribution of nursing coordination as an exclusive task of nurses
7.	Search for strategies to improve teaching quality
8.	Re-elaboration of nursing curriculum

The participants indicate that several institutions have supported and established a curriculum revision, in the attempt to change the focus to primary care. In addition, the new proposed curriculum should cover expected nursing competencies to work in care, teaching, management and research.

## Discussion

The challenges addressed are not exclusive to the nursing area. A study developed in 20 countries, including Angola, emphasizes the lack of health professionals to respond to the population needs [11], which demonstrates the importance of education and the continuous development of these resources. In another recent study, involving medical students enrolled in public schools in Angola, Guinea-Bissau and Mozambique, which was aimed at describing and analyzing expectations about the profession and the difficulties related to education and the professional future, it was identified that investments in the infrastructure and preparation of higher education institutions are fundamental to achieve institutional success.

Particular attention needs to be devoted to students’ socialization and teachers’ roles and status. In countries with a lack of resources, a focus on the hospital area is noticeable, instead of being associated to skills development for work in primary care areas. The development of local graduate programs can serve as an interesting strategy to support the retention of professionals in their country of origin [12].

As regards the valuation of nurses, an incipient political movement seems to be on course to acknowledge nursing as an important part of health services. In a speech on the occasion of the Day of the Nurse, celebrated in May 2012, the Minister of Health of Angola, Mr José Van-Dúnem, recalled that nurses are responsible for controlling and guaranteeing therapy and for transmitting moral and psychological support to patients and their families. He added that nursing can get closer to the community and enhance the effective execution of municipal health programs, so as to reduce the demand referral hospitals are confronted with and return the population’s confidence in health professionals. According to an article published in *Jornal de Angola*, another aspect the minister highlighted was the improved efficiency and quality of health services and the valuing of human beings [13].

In 2012, the Angolan Nursing Council chose the theme "Fighting inequality: from evidence to action”. According to the Minister of Health, the theme follows the executive power’s concern with making efforts to improve work conditions and serve as motivation for nursing professionals to continue working with their characteristic zeal and dedication [13].

The proposal disseminated by the Angolan Nursing Council, as presented in the Activity Plan and Budget for 2011, underlines concerns with nursing education, in accordance with the participants’ reports. In the document, the need to strengthen the qualified interventions of educational policiesis highlighted, including proactive participation in the redesign of organized responses to the learning needs. As fundamental pillars for the autonomic and developmental process of the profession, education and research continue to be in need of further professional and political deepening [14].

In other African countries, difficulties for nursing education are similar. In Kenya, the problematic situation of higher education, mainly related to the characteristics of the education system in force and the lack of public support, does not favour educational opportunities after gaining a basic degree. The country’s experience has shown that distance education is effective and can support professional nursing training at different levels, including leadership in clinical practice, teaching, administration and research [15].

South Africa’s experience shows a legacy of inequalities in health care access, growing morbidity and mortality rates and an insufficient number of qualified nurses to achieve the MDGs the government defined in 2010. According to researchers, nurse managers need to influence political decisions that involve nursing services and the education needed to prepare the next generation of professionals to execute these new services [16].

In Ghana, to solve problems related to health service delivery and the lack of nursing professionals, the participation of all stakeholders was required, moving beyond traditional stereotypes, showing flexibility and orientation towards the future. Political options included the expansion of nursing education, broadening of teaching locations, offering of professional training, and collaborative education opportunities, which improved personal satisfaction rates and professionals’ continuation in their country. The international exchange of nurses and resources was shown to be mutually beneficial for the countries of origin and the collaborators [17].

The convergence between nursing education difficulties in Angola and other African countries, as well as the solutions proposed to minimize the problems raised, show the urgent need to elaborate and put in practice strategies to improve the quality of education and professional teaching and practice conditions in nursing, leading to the acknowledgement of the role of nursingin the context of the health-disease and teaching-learning process, among others. The Brazilian researchers discussed the fact that all issues addressed demand paradigm changes in professional education, with a view to the collective construction of a more active and committed nursing workforce, which participates in decision-making on public, social and institutional policies. The same discussion and approach can be directed at African countries such as Angola [18].

The Ouagadougou Declaration on primary health care and health systems in Africa,*Achieving Better Health for Africa in the New Millennium*, provides a framework for implementing necessary activities in each of the WHO priority programme areas. The Declaration proposes recommendations for consideration by Member States in the development of their own country frameworks. All 46 Member States have national health sector development policies and plans, which provide a platform for the national health agenda. A number of countries have developed HRH policies and plans [19].

The predominance of male participants stands out in the sample. Angola has a 27-year history of civil war, which started in 1975, caused by a global identity crisis that was associated with exogenous factors. The conflict originated countless internal and external migration movements. A mass migration movement was observedin search of new existential possibilities, particularly among the male population in response to fears of combat and the consequences of civil war [20]. The literature about the theme is scarce, but the participants’ reports revealed that most men who emigrated looked for educational opportunities in other countries, assuming the commitment to return home with better social, cultural and financial development conditions for their families and compatriots. This can justify the predominance of male participants in the population of nurse leaders.

To date, many African health professionals have migrated to developed nations to promote their education and career, leading to a lack of health professionals in their countries of origin. The Organization of Economic Cooperation and Development (OECD) estimates that 18% of all physicians and 11% of all nurses who work in their countries are foreigners. This migration aggravates the lack of health professionals and contributes towards the weakening of poor nations’ health systems, which have failed in their attempts to manage the severe health problems that affect the population [21].

According to Mateus Foster, the director of international issues of the Royal College of Physicians in London, the reasons for this migration go beyond the very small remuneration and involve the work and living conditions, education and, at the bottom, continuous professional development [21].

Nursing in Angola has reached a moment to conquer its professional space. Therefore, it is fundamental to establish regulatory policies, defining the rights and duties of all categories involved, to determine and legitimize nurses’ function in the health team, including respect for and acknowledgement of their role in the community [22].

The African Region continues to focus on standardizing guidelines and curricula and is actively implementing competence-based training for nurses and midwives, from pre-service through to faculty education levels. Despite challenges, progress is being made. Some countries are actively implementing the strategies to enhance institutional capacity and the education of suitably skilled practitioners. Scaling up the strengthening of nursing and midwifery education is accelerating in all six WHO. Curriculum evaluations for pre-service nursing and midwifery education programmes have been conducted in West and East African countries. Challenges identified include: curricula that are not competence-based; unclear distinctions between nursing and midwifery especially in francophone countries; shortage of teachers; and inadequate training and teaching materials and infrastructure [19].

In addition to the fact that not all leaders contacted were able to attend, another study limitation is that it cannot be guaranteed that the participants demonstrated their complete potential to contribute to the discussion fulltime. Also, as the data collection was formally organized and involved acquaintances, there may have been concerns about what should or should not be said. When analyzing the results, it would be interesting to combine the data with other information obtained through different data collection techniques, which goes beyond the present research objectives [23].

Also, the statistical database available about Angola is relatively outdated and very fragile. Despite the pertinence of the sources used, it is important to indicate some limitations in the data, due to the lack of studies and published material about the theme.

## Conclusion

The results of this study have demonstrated that technical and vocational schools in nursing in Angola face many challenges, related to the lack of infrastructure, absence of trained human resources especially in the public sector, bureaucratic problems to regularize schools and lack of material resources.

On the other hand, the solutions they present are predominantly focused on the valuing of nursing professionals, which implies cultural and attitudinal changes. In that sense, public health education policies in Angola should establish action guidelines that permit nurses’ effective activities, including the regularization of vocational schools and based on the recognition of nurses as key elements to enhance the quality of health services in that country.

## Abbreviations

WHO: World Health Organization; HDI: Human development index; MDG: Millennium development goals; PALOP: Portuguese-speaking African countries; ETPS: Technical and Vocational Schools in Health; HEI: Health Education Institution; IMES: Secondary Education Institutes in Health; OECD: Organization of Economic Cooperation and Development.

## Competing interests

The authors declare that they have no competing interests.

## Authors’ contributions

LMM-A, IACM and CAV elaborated the concept and design of the study, drafted the manuscript and were responsible for the critical revisions regarding the content. LMM-A and AM were involved with data collection and building the databank, data interpretation and analysis, and drafting the manuscript. MAT and SG worked on building the databank, protocol design and data analysis, and were responsible for administrative and technical support. All authors read and approved the final version of this manuscript.

## Authors’ information

The authors’ work in Angola was executed in the framework of a project the Global Network of WHO Collaborating Centres for Nursing and Midwifery Development executed under the leadership of IACM and CAV.
